# Supplementary material for: CREaTor: zero-shot cis-regulatory pattern modeling with attention mechanisms
Source: Genome Biol. 2023 Nov 23;24:266. doi: 10.1186/s13059-023-03103-8 (PMC10666311; doi:10.1186/s13059-023-03103-8)
Supplement: Supplementary file 1 — Additional file 1: Fig. S1. Architecture of CREaTor. Fig. S2. Data split strategy for CREaTor Training and evaluation. Fig. S3. Prediction of K562 differentially expressed genes. Fig. S4. UMAP visualization of 123 expression profiles. Fig. S5. Leave-one-chromosome-out and leave-one-cell type out assays. Fig. S6. Performance of CREaTor with cCREs up to 2kb, 5kb, 10kb, 100kb, or 1Mb away from the TSS of target genes. Fig. S7. Statistics of enhancer-gene interaction data from 3 CRISPRi-based studies. Fig. S8. auPRC and auROC of CREaTor and its counterparts on the classification of cCRE-gene pairs collected from 3 independent CRISPR perturbation experiments. Fig. S9. Specificity and precision scores of CREaTor and its counterparts on cCRE-gene pair classification. Fig. S10. Average signals of RNA Pol II on cCREs in cluster 0 and cluster 1 respectively. Fig. S11. Gene representations learned by CREaTor can be clustered into groups with different functions. [file 13059_2023_3103_MOESM1_ESM.docx]

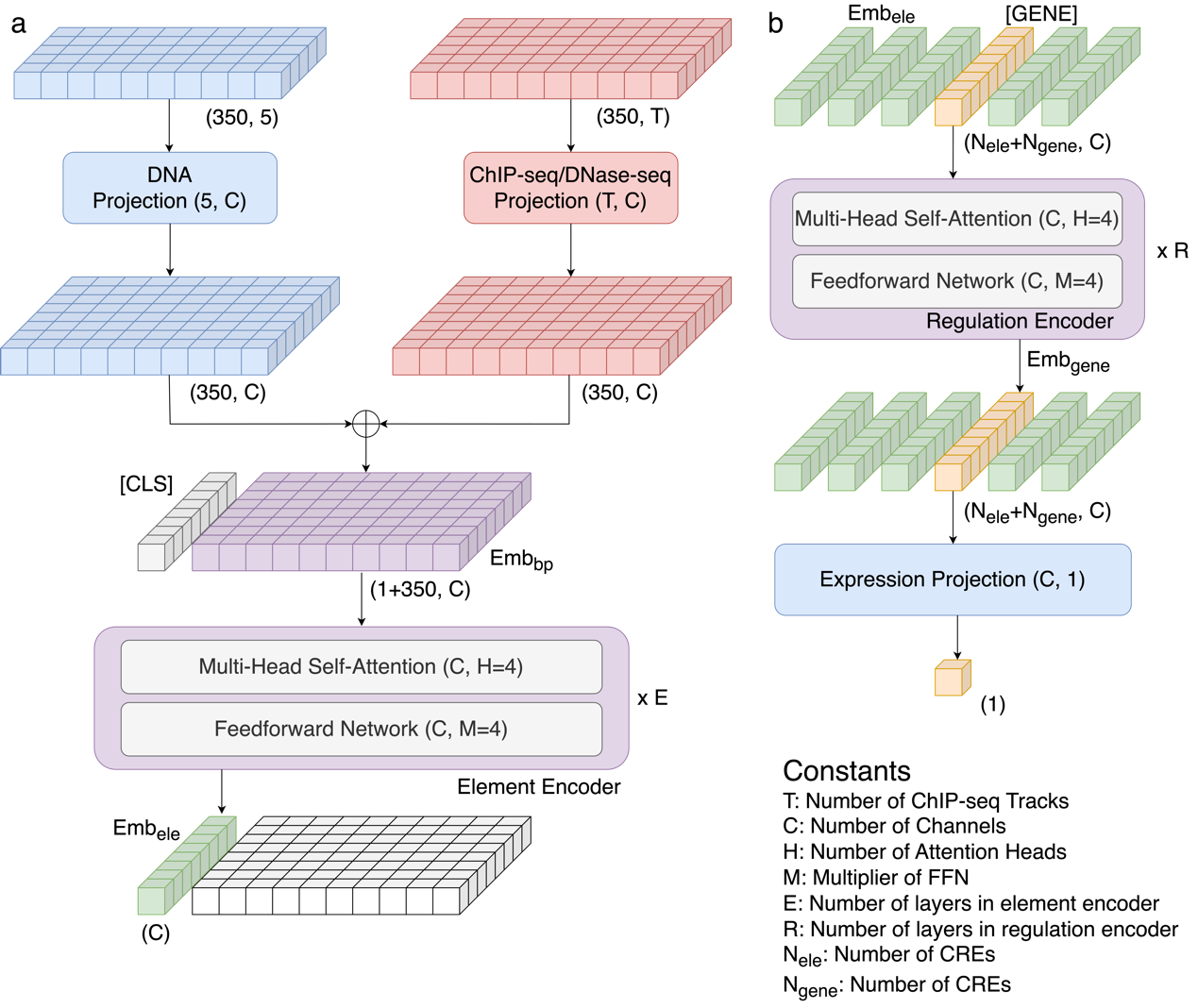


**Fig S1: Architecture of CREaTor.** CREaTor is composed of two modules. **a)** Element module encodes the representation of cCREs. We first map DNA and ChIP-seq/DNase-seq to latent space through a linear projection respectively, and then combine them through element-wise addition to obtain $Emb_{bp}$, the feature embedding of each bp. We feed the $Emb_{bp}$s into the element encoder together with a [CLS] token. The [CLS] token adaptively aggregates information from the $Emb_{bp}$s in the element encoder. We use the output vector of [CLS] token as the representation of the element, namely $Emb_{ele}$. **b)** Regulation module models the interaction between cCREs and genes. We concatenate the $Emb_{ele}$ of cCREs (denoted in blue and yellow) and the [GENE] tokens (denoted in red) as the input of the regulation encoder. The [GENE] tokens interact with and are regulated by the cCREs in the regulation encoder. We apply a linear projection with SoftPlus activation on the output vector of [GENE] tokens to predict their expressions. The size of each component of the architecture is shown as a tuple inside the block. The shape of the tensor at each step is denoted as a tuple in the bottom right of the blocks.


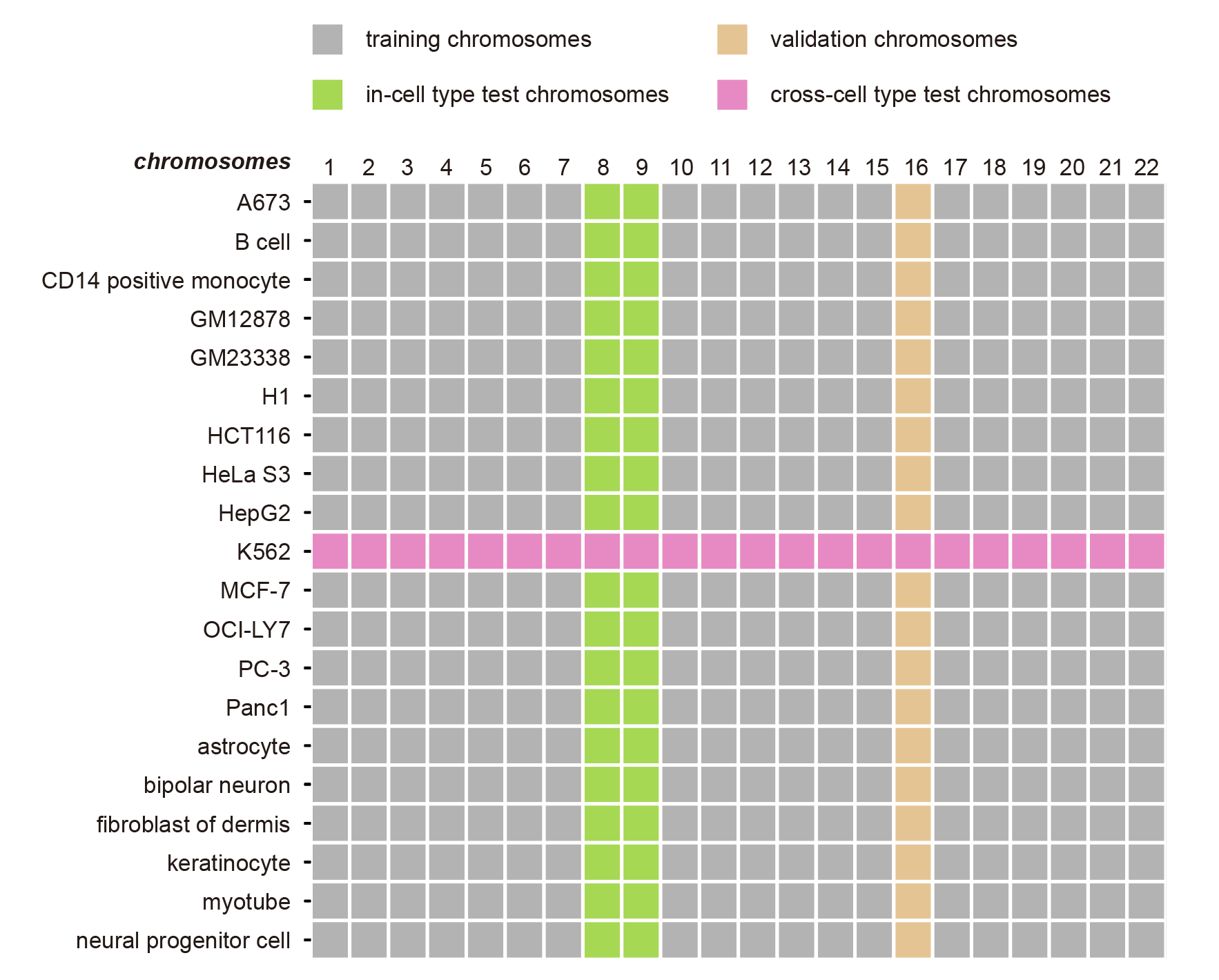


**Fig S2:** **Data split strategy for CREaTor Training and evaluation.** Training chromosomes (grey): chr1-7, chr10-15, and chr17-23 of 19 human tissues and cell lines: A673, B cell, CD14 positive monocyte, GM12878, GM23338, H1, HCT116, HeLa S3, HepG2, MCF-7, OCI-LY7, PC-3, Panc1, astrocyte, bipolar neuron, fibroblast of dermis, keratinocyte, myotube, and neural progenitor cell. Validation chromosomes (khaki): chr16 of the same 19 human tissues and cell lines. Additionally, we designed 2 sets of test datasets: In-cell type test chromosomes (green) denote chr8 and chr9 in the 19 human tissue and cell lines (excluding K562). Cross-cell type test chromosomes (magenta) denote all autosomes of K562 cell line, as K562 is not used for model training.


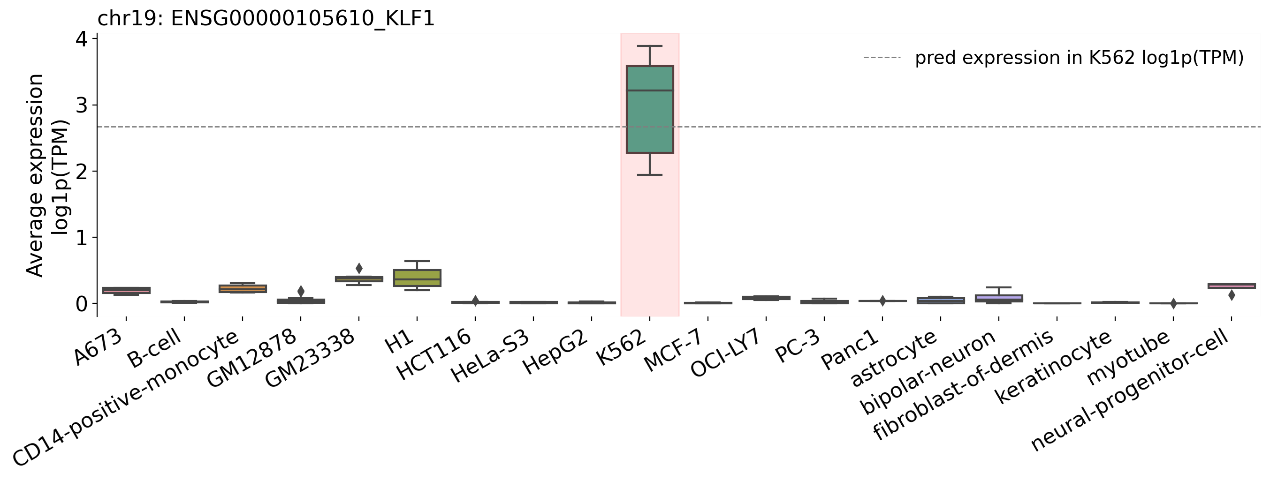

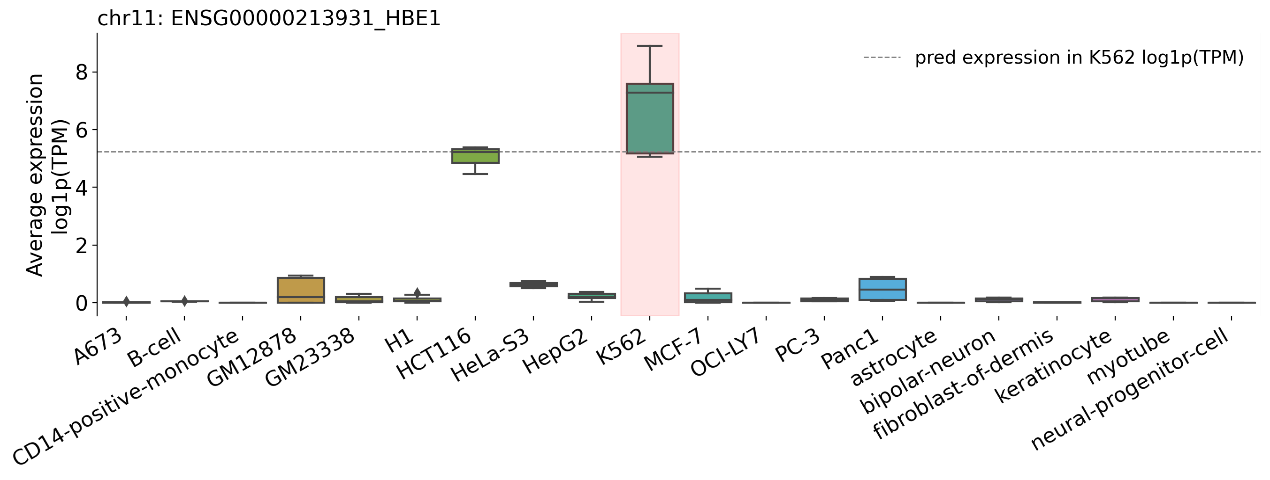

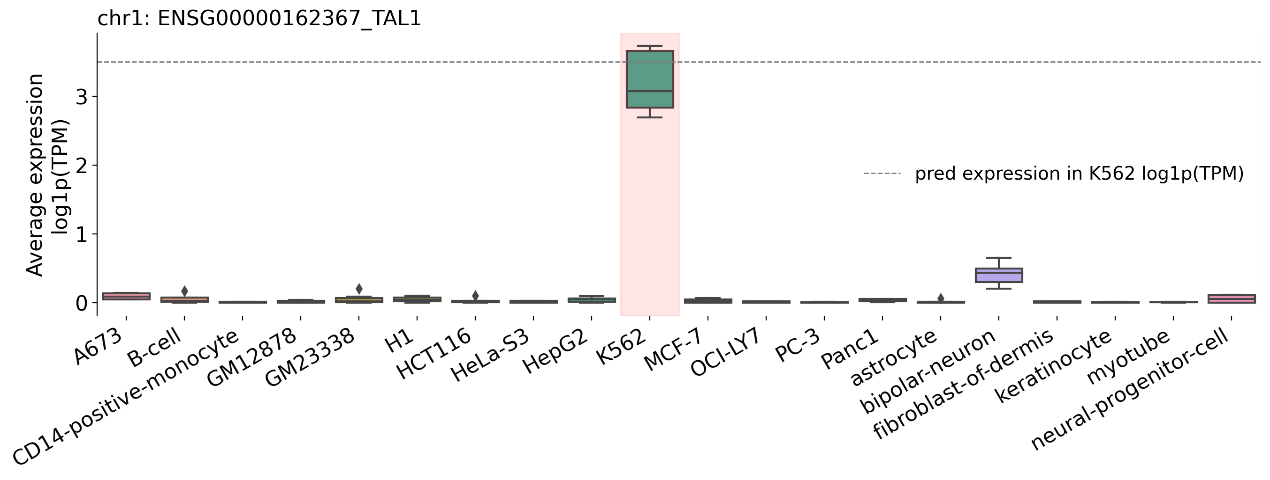


**Fig S3:** **Prediction of K562 differentially expressed genes.** Representative examples of observed and predicted expressions of genes KLF1, TAL1 and HBE1 in 20 different types of cells. The dashed line indicates the predicted values.


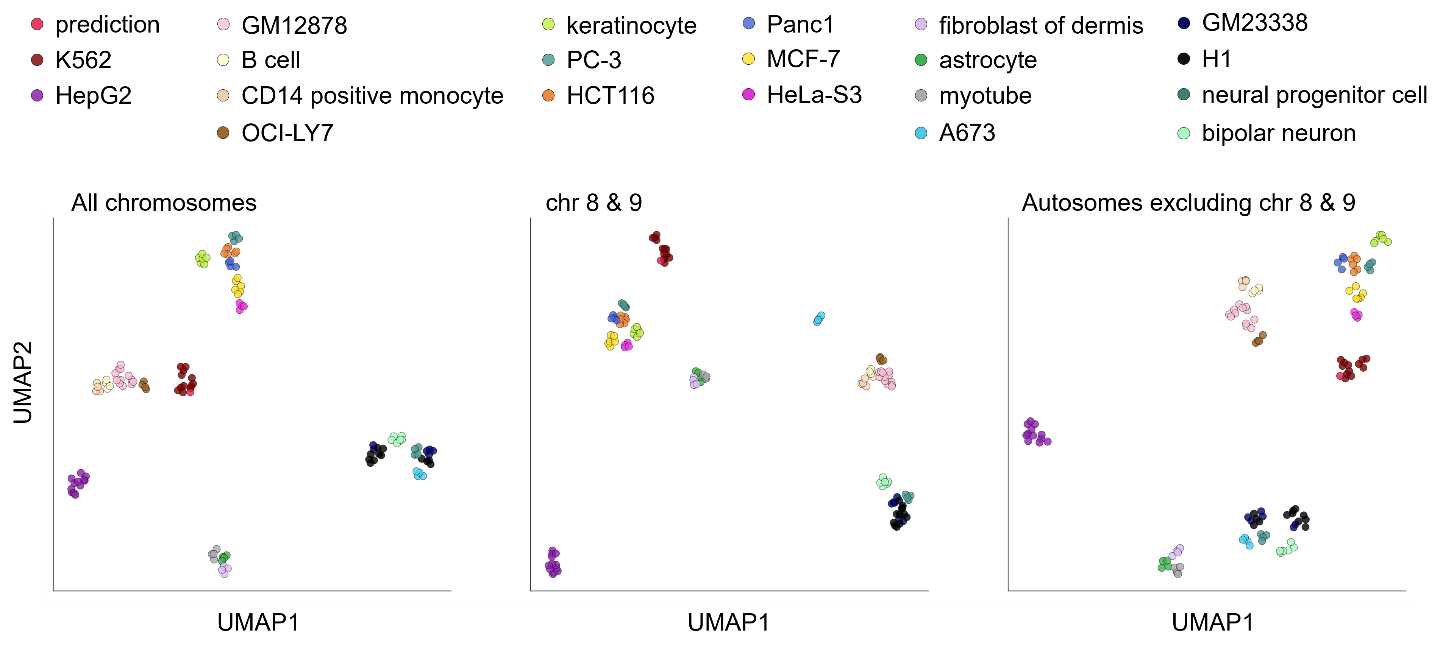


**Fig S4:** **UMAP visualization of 123 expression profiles.** Prediction: Predicted K562 gene expression profile. Others: Transcript quantifications of corresponding cell types with RNA-seq.

**
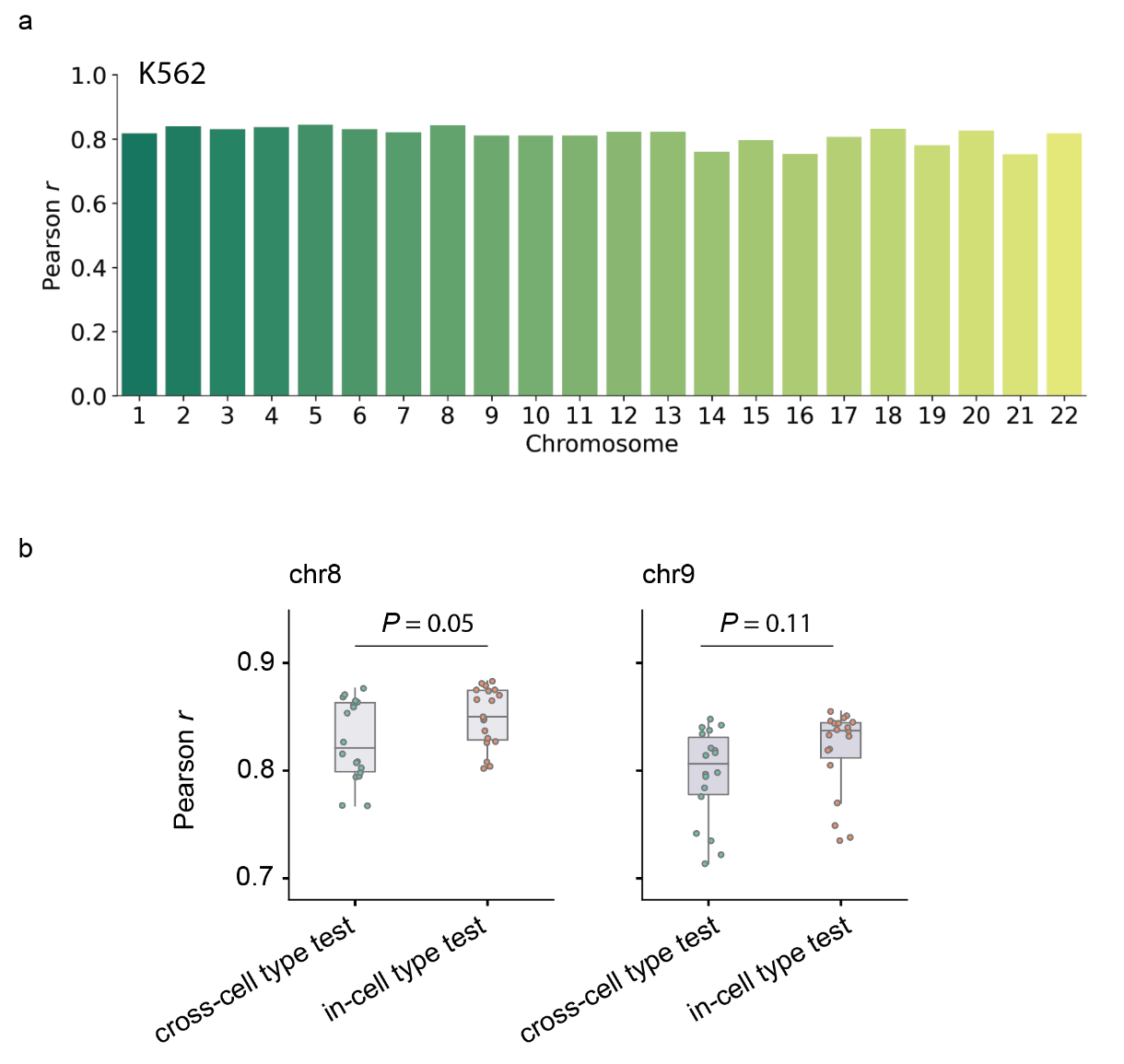
**

**Fig S5:** **Leave-one-chromosome-out and leave-one-cell type out assays.** **a)** Pearson *r* between predicted and observed expression of genes on chr1-22 in K562. In this leave-one-chromosome-out assay, each chromosome was excluded from training once, and the corresponding chromosome in K562 was used for test (Additional file 2: Table S4a). **b)** Pearson *r* between predicted and observed expression of genes on chr8 and chr9 in different cell types under different settings. Cross-cell type test: as in the leave-one-cell type-out setting, all chromosomes of each cell type were held out for test once (Additional file 2: Table S4b). In-cell type test: The cell type was used for modeling training, but chr8-9 were held out for test (Additional file 2: Table S2). Each dot represents one cell type. P values were computed with the two-sided Mann–Whitney U test.


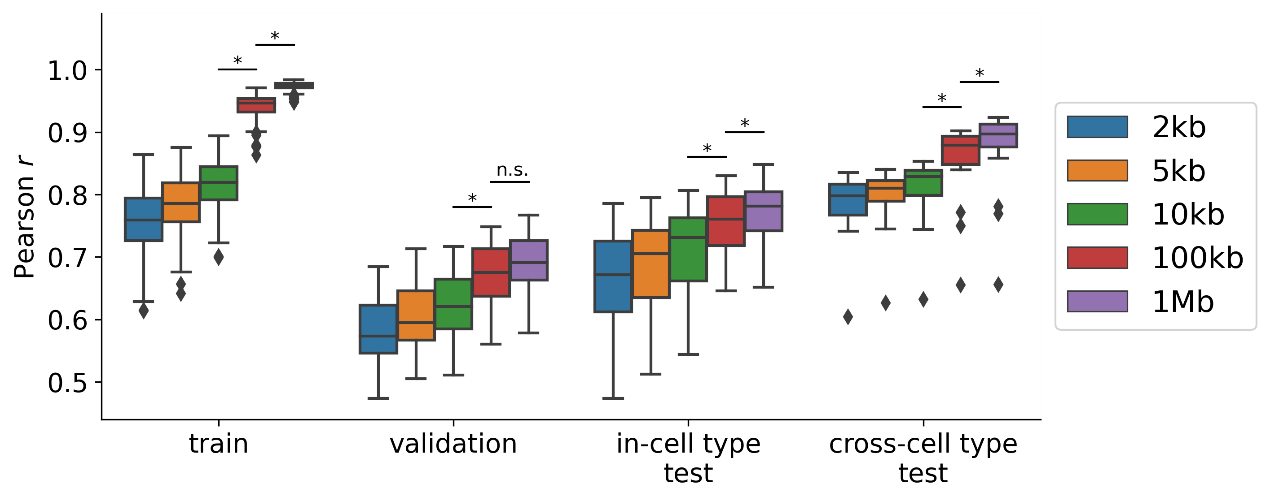


**Fig S6:** **Performance of CREaTor with cCREs up to 2kb, 5kb, 10kb, 100kb, or 1Mb away from the TSS of target genes.** The training set includes chr1-7, 10-15, and 17-22 in 19 cell types other than K562. The validation set includes chr16 in cell types other than K562. The in-cell type test set includes chr8 and chr9 in cell types other than K562. Cross-cell type test set represents all chromosomes in the K562 cell line. P values were computed with the two-sided Mann–Whitney U test.


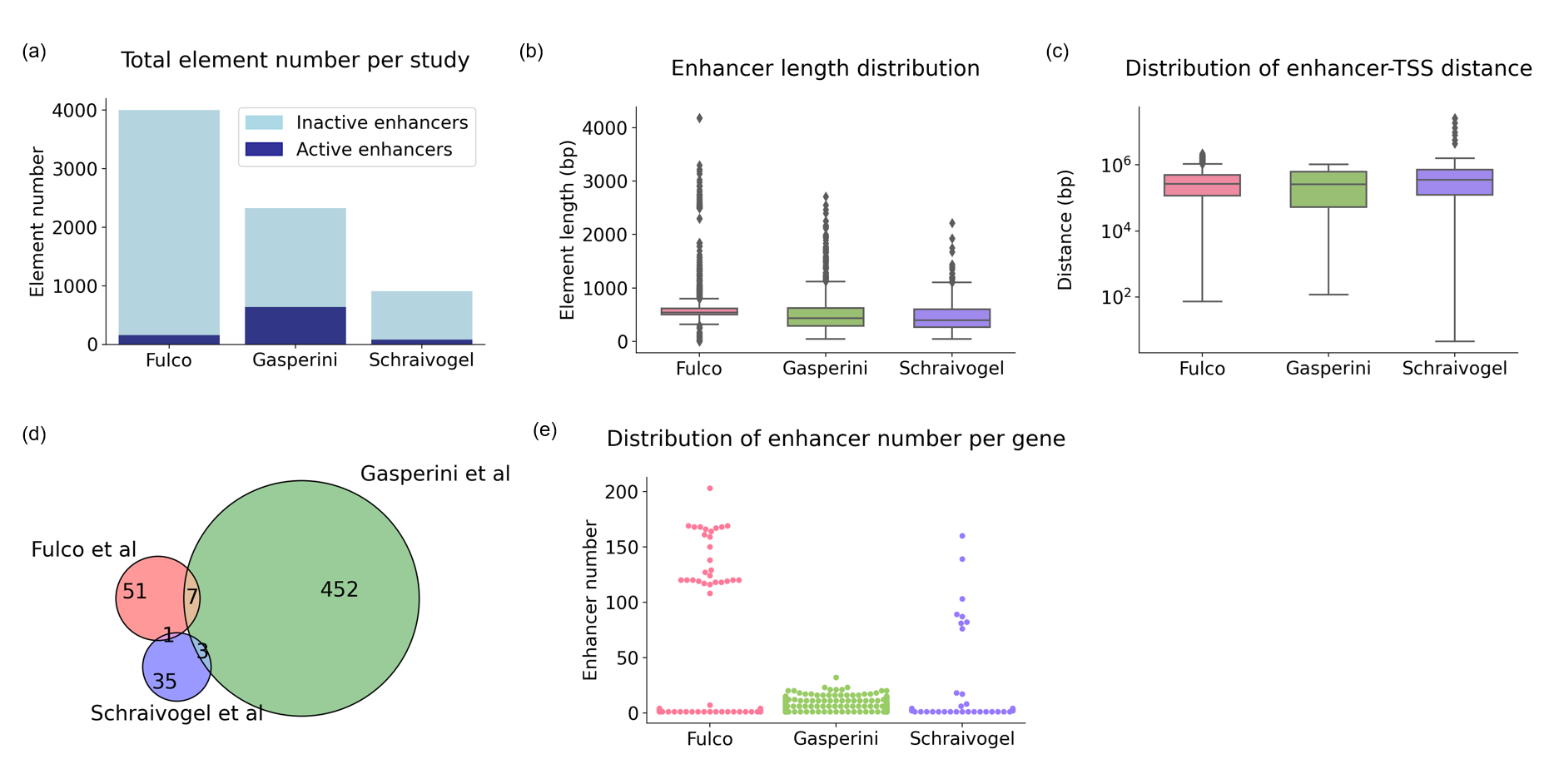


**Fig S7: Statistics of enhancer-gene interaction data from 3 CRISPRi-based studies.** The statistics were performed on data after genomic coordinates liftover and non-autosomosal data filtering. **(a)** The number of active and inactive enhancers tested by each study. **(b)** Enhancer length distribution in each study. **(c)** Enhancer-gene TSS distance distribution in each study. **(d)** Overlapped active enhancers in 3 studies. **(e)** The number of enhancers tested for each gene in each study.


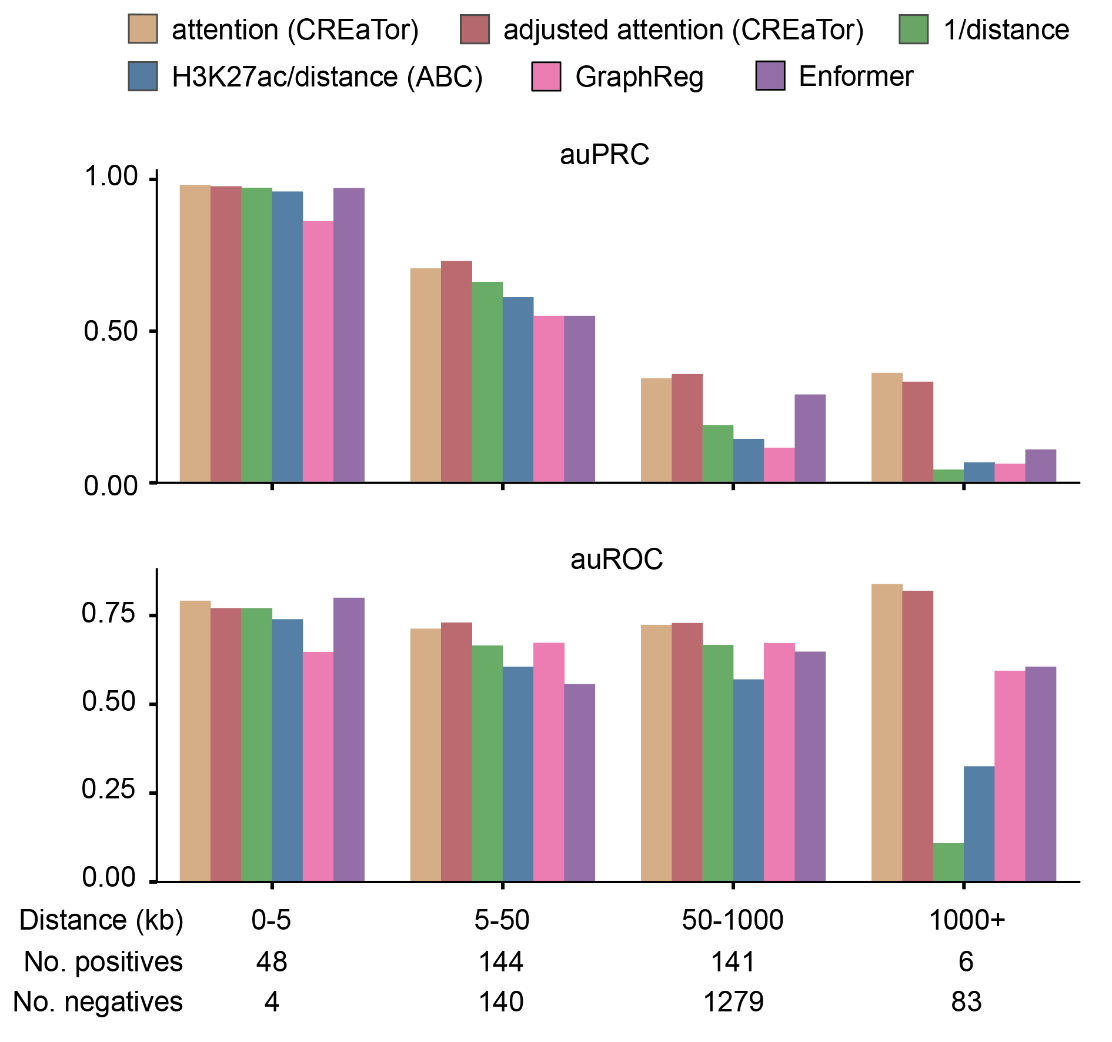


**Fig S8: auPRC and auROC of CREaTor and its counterparts on the classification of cCRE-gene pairs collected from 3 independent CRISPR perturbation experiments.** cCRE-gene pairs are stratified by their relative genomics distances. The number of positive/negative labels in each group is annotated at the bottom. Labels (positive/negative) of cCRE-gene pairs were collected from CRISPR perturbation experiments.


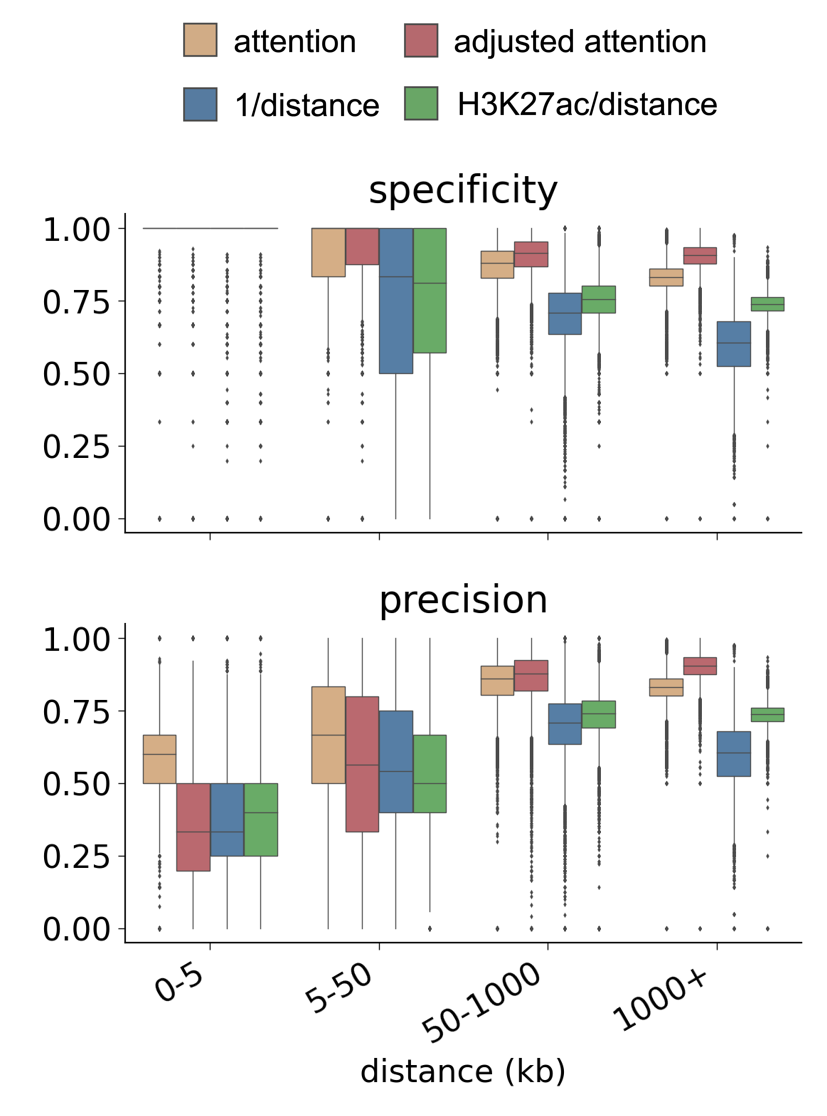


**Fig S9: Specificity and precision scores of CREaTor and its counterparts on cCRE-gene pair classification.** Distance denotes the relative genomic distance between cCREs and genes. The performance is evaluated for each gene and each distance group separately. The H3K27ac value of a cCRE is calculated as the sum of the H3K27ac peak values of the element. Positive/negative cutoff is set as mean values of attention scores in each distance group. Labels (positive/negative) of cCRE-gene pairs were collected from a Pol-II mediated ChIA-PET experiment of K562.

**
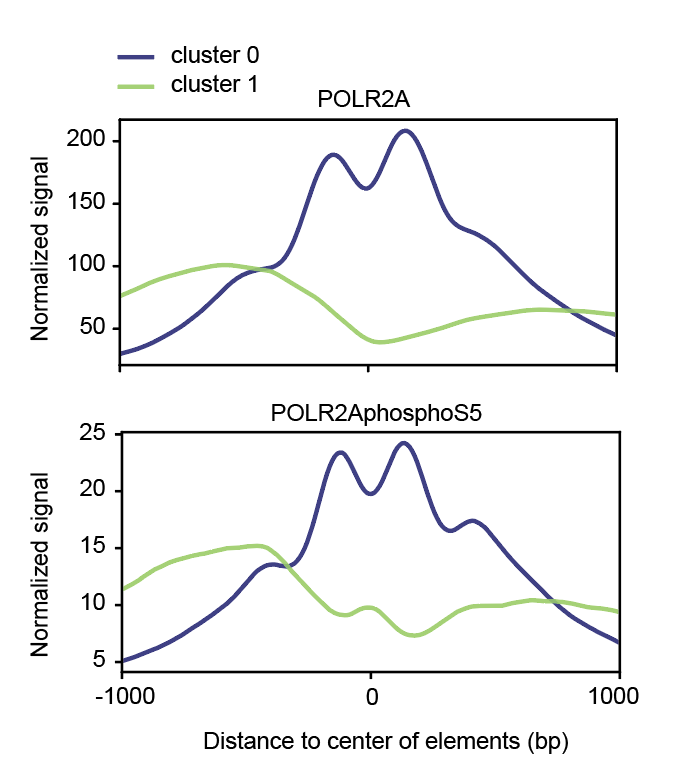
**

**Fig S10: Average signals of RNA Pol II on cCREs in cluster 0 and cluster 1 respectively.** Upper: unphosphorylated form of Pol II. Bottom: Pol II CTD phospho Ser5.


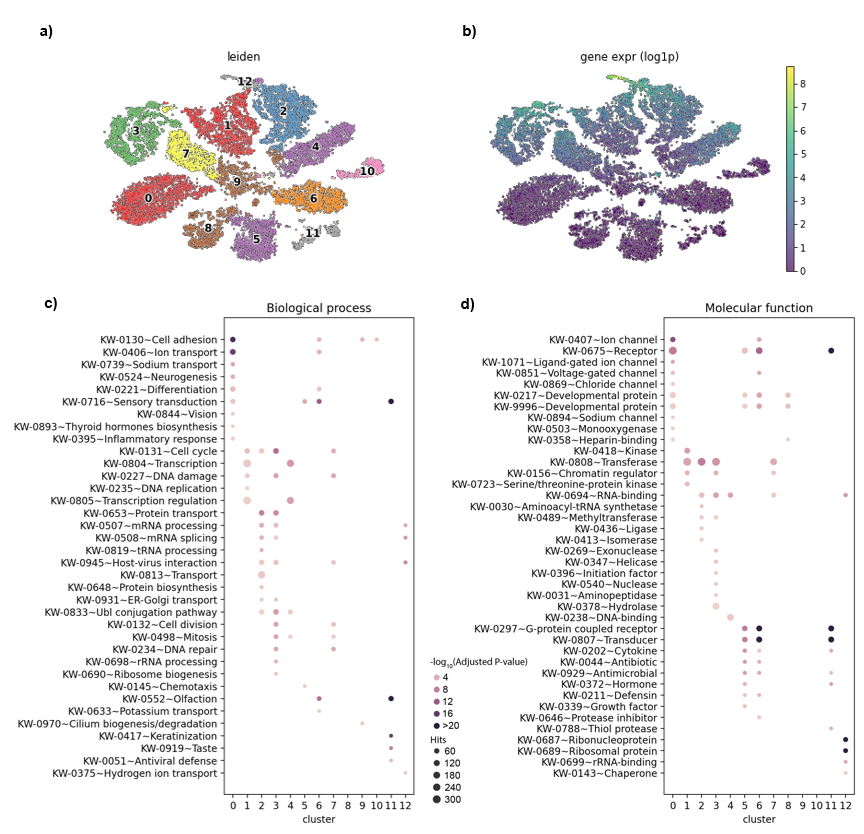


**Fig S11: Gene representations learned by CREaTor can be clustered into groups with different functions. a)** Uniform Manifold Approximation and Projection (UMAP) of gene embedding in K562, colored and numbered as clusters grouped by the Leiden algorithm. **b)** Same as (a), but colored by gene expression levels. **c)** Functional annotation clustering with the DAVID Gene Functional Classification Tool (DAVID, http://david.abcc.ncifcrf.gov) using UniProtKB biological process keywords. Significantly enriched (adjusted p-value<0.05) groups for genes in each cluster in (a) are shown. **d)** Functional annotation clustering with DAVID using UniProtKB molecular function keywords. Significantly enriched (adjusted p-value<0.05) groups for genes in each cluster in (a) are shown.
