# Supplementary material for: CREaTor: zero-shot cis-regulatory pattern modeling with attention mechanisms
Source: Genome Biol. 2023 Nov 23;24:266. doi: 10.1186/s13059-023-03103-8 (PMC10666311; doi:10.1186/s13059-023-03103-8)
Supplement: Supplementary file 4 — Additional file 4. Review history. [file 13059_2023_3103_MOESM4_ESM.docx]

**1st round**

**Reviewer 1**

The authors present a new deep neural network model for predicting gene expression from both genomic DNA and local epigenomic context. The model is evaluated on prediction of gene expression on left out regions, and further the model is dissected to study the relationship between DNA grammar and gene regulatory mechanisms that specify cell-type specific gene expression. The main novelty is potentially the use of a new transformer architecture to more accurately model the joint impact of epigenomic and DNA sequence features on gene expression. However, as I explain below, I have significant concerns below regarding model evaluation and novelty.

First, I find the results hard to interpret due to the lack of coherent description of how experiments where performed, what data was used, and how the models where evaluated. Thus, I am unsure if the reported performance can be meaningfully interpreted, which impacts the ability to evaluate the later model interpretation results.
- For example, the authors claim that CReaTor results in prediction correlation accuracy of ~0.8-0.9 on unseen chromosome 8-9. This results should be summarized in Table Extended S1, but instead Table ExtendedS1 is reporting performance on every chromosome. Some of these are training data? There are no table legends that clearly describe what the tables are showing.

- Similarly, I can't find which cell types where left out for which performance. If a left out cell lines/type is similar to the ones in the training data, there is no surprise that the performance on "un seen" cells will be similar to seen cells, therefore, to meaningful report this sort of performance, one needs to also consider the correlation between the unseen cell and the "cells" in the training data. Finally, evaluating the performance on one unseen cell or two left out chromosomes is not sufficient. A proper "leave-out-one-at-a-time" approach is needed, where performance is averaged across several left out chromosomes and cell types (ideally each chromosome left out once and results are separately shown, so. that the reader can assess variability in performance. A similar approach should be take for evaluating generalizability to unseen cell types).

A second major comment is the lack of model comparison with previous approaches. For example, MaxATAC (PLoS CompBio 2023), GraphReg (Genome Research 2022), and ChromDragoNN (Bioinformatics 2019) are three models that use a similar concept: they all use as input both DNA sequence and epigenomic features and aim to predict gene expression. It's unclear what the novelty is here. If the novelty is in the model architecture, then the new architecture should be benchmarked against these previous methods.

**Reviewer 2**

In this paper, Li et. al. introduce a hierarchical deep neural network, named CREaTor, developed to predict interactions between candidate cis-regulatory elements (cCREs) and their target genes. The network incorporates an attention mechanism to model interactions between genomic elements, even those located 2Mb apart, thereby providing a more detailed representation of gene regulation involving cis-regulatory programs. In an evaluation using a distinct cell type, the authors demonstrate that CREaTor surpasses several of the previously existing methods in encapsulating interactions between cCREs and genes over differing distance ranges.

Overall, I enjoyed reading this paper. The real meat of this study is the innovative, well-developed tool that shows promise in overcoming long-standing challenges in regulatory genomics. The authors make a compelling case that functional data is likely to help CREaTor generalize to new cell types better than models trained using DNA sequence alone, and hence I can see this overall strategy becoming widely deployed for the purposes argued by the authors. The writing is clear (though there are multiple typos and grammatical errors that need to be addressed).

I have several comments that should be addressed prior to publication:

* The authors seek to draw conclusions about the importance of proximal regulatory sites based on findings that the model maintains substantial predictive power when trained on windows just 2kb away from the gene. However, much of distal regulation occurs at enhancers within the gene itself, but far away from the gene's promoter. To really support the author's conclusion as stated, the author should train models using data from just 2kb away from the transcription start site.

* The author should probably benchmark against the other attention-based enhancer-promoter prediction engine published recently, Enformer (Avsec et. al. 2021).

* The final two browser tracks in Fig 2 have significant rendering issues.

* The authors write that: "attention weights of CREaTor and quantitative effects of enhancers on gene expression measured by a CRISPR perturbation study13 yielded a spearman ρ of -0.269 (Fig. 2b), implying that CREaTor may capture quantitative effects of cCREs to genes." It is not intuitive that the correlation should be negative. The author should clarify here and elsewhere that the measurement they call "effects of enhancers on gene expression" is actually the effect of enhancer knockdown (and hence, the enhancer would presumably have the opposite effect).

**Authors’ response**

We have read reviewers’ comments carefully and made revisions accordingly in the  manuscript (highlighted in red). Meanwhile, we addressed all the concerns of the reviewers in the point-by-point response letter below. Briefly,

1) To address Reviewer 1's concerns regarding the novelties and highlights of our study, we have modified the title, abstract, background (Line 57-61 and Line 68-76), and  discussion (Line 329-333) of the manuscript to clarify the purpose of our study.

2) In response to Reviewer 1's concerns about model interpretations and evaluations,  we added Supplementary Figure 1 to illustrate our data split strategy, conducted two  sets of Leave-one-out experiments (Supplementary Table 2 and Supplementary  Figure 3), analyzed expression profile similarities (Supplementary Figure 2), and  updated the manuscript accordingly.

3) To address Reviewer 1 and Reviewer 2's concerns about the lack of comparisons, we  compared our method with 2 SOTA approaches (Enformer and GraphReg) with additional experiments. Details regarding Enformer and GraphReg implementations are described in both the point-by-point response letter and the Methods section of  the manuscript. We also elaborated on the reasons for not considering the 2 alternative approaches proposed by the reviewers in our response letter.

4) In response to Reviewer 2's comments on typos, unclarities, and rendering issues in  the figures, we have carefully reviewed the manuscript and corrected the relevant  flaws.

Reviewer #1: ===

*The authors present a new deep neural network model for predicting gene expression from  both genomic DNA and local epigenomic context. The model is evaluated on prediction of  gene expression on left out regions, and further the model is dissected to study the  relationship between DNA grammar and gene regulatory mechanisms that specify cell-type  specific gene expression. The main novelty is potentially the use of a new transformer  architecture to more accurately model the joint impact of epigenomic and DNA sequence  features on gene expression. However, as I explain below, I have significant concerns below  regarding model evaluation and novelty.*

We thank the reviewer for the critical comments and constructive suggestions. We  acknowledge the concerns raised and have addressed them by making adjustments to the  manuscript and conducting additional experiments as needed.

Your comments are laid out in *italicized, blue* font, and specific concerns have been  numbered. Our responses are given in regular font, and changes/additions to the manuscript  are highlighted in red.

A brief summary of the concerns and focus of responses can be found below:

In response to *Q1*, we have clarified the **settings** used for model training and evaluation, for  both gene expression prediction and cCRE-gene interaction modeling.

For *Q2*, we performed **additional experiments** to showcase the **generalization** ability of  CREaTor in predicting gene expression across different cell types.

Regarding *Q3*, we have emphasized the **novelty** of our methods and provided **comparisons  with previous approaches**, highlighting the advantages of CREaTor, such as its  generalization ability, interpretability, and applicability to various scenarios.

We believe that these revisions and additional experiments have addressed the reviewer's  concerns, and we hope that the updated manuscript better demonstrates the significance  and potential of CREaTor in the field of gene regulation research.

*Q1. First, I find the results hard to interpret due to the lack of coherent description of how  experiments where performed, what data was used, and how the models where evaluated.*

*Thus, I am unsure if the reported performance can be meaningfully interpreted, which  impacts the ability to evaluate the later model interpretation results.*

*- For example, the authors claim that CReaTor results in prediction correlation accuracy  of ~0.8-0.9 on unseen chromosome 8-9. This results should be summarized in Table  Extended S1, but instead Table ExtendedS1 is reporting performance on every chromosome.  Some of these are training data? There are no table legends that clearly describe what the  tables are showing.*

Response:

We apologize for the misleading and unclear description of experimental settings. Here, we  1) described detailed settings we used for model training and evaluation, and 2) listed the  improvements and corrections we made to the manuscript.

Our study aims to model cis-regulatory patterns and cCRE-gene interactions with improved  accuracy in new cell types without fine-tuning orretraining. To achieve this goal, we designed  a training task to predict gene expression based on flanking candidate cis-regulatory  elements (cCREs) up to 2Mb from target genes.

For the task of gene expression prediction, which is discussed in Figure 1 and *CREaTor  predicts cell type-specific gene expression in unseen cell types* part of the Result Section:

• The model is ***trained*** with **chr1-7, chr10-15, and chr17-23 in 19 human tissues and  cell lines**: A673, B cell, CD14 positive monocyte, GM12878, GM23338, H1, HCT116,  HeLa S3, HepG2, MCF-7, OCI-LY7, PC-3, Panc1, astrocyte, bipolar neuron, fibroblast  of dermis, keratinocyte, myotube, and neural progenitor cell.

• The model is ***validated*** with **chr16 in the same 19 human tissues and cell lines**. Additionally, we designed 2 sets of test datasets:

• ***In-cell type test chromosomes*** denote **chr8 and chr9 in the 19 human tissue and  cell lines** (excluding K562), and the results are reported in Extended Data Table 1 and  Fig. 1c (left).

• ***Cross-cell type test chromosomes*** denote **all autosomes of K562 cell line**, as  K562 is not used for model training. The results of cross-cell type test chromosomes  are reported in Extended Data Table 2 and Fig. 1c (right).

We define K562 autosomes as cross-cell type test chromosomes because K562 has  the most abundant labeled enhancer-gene pair data, which are used to evaluate the  model’s performance on the major task, enhancer-gene interaction prediction, of our  study.

The description of the setting can be found at Line 97-105 of the manuscript, as well as in Fig.  1b. To present the information more clearly, we

• Added table legends to Extended Data Table 1 and Extended Data Table 2. • Added an elaborate illustration of the setting in Supplementary Figure 1.

For the task of cCRE-gene interaction prediction, which is discussed in Figure 2 and *Self attention reveals functional cCREs in unseen cell types* part of the Result Section:

• No training was performed.

• We curated 2 evaluation datasets from 1) 3 independent CRISPR perturbation studies  (Fulco et al., 2019; Gasperini et al., 2019; Schraivogel et al., 2020) and 2) a genome wide Pol II-mediated ChIA-PET dataset (ENCSR880DSH).

*Q2. Similarly, I can't find which cell types where left out for which performance. If a left out  cell lines/type is similar to the ones in the training data, there is no surprise that the  performance on "un seen" cells will be similar to seen cells, therefore, to meaningful report  this sort of performance, one needs to also consider the correlation between the unseen cell  and the "cells" in the training data. Finally, evaluating the performance on one unseen cell or  two left out chromosomes is not sufficient. A proper "leave-out-one-at-a-time" approach is  needed, where performance is averaged across several left out chromosomes and cell types  (ideally each chromosome left out once and results are separately shown, so. that the reader  can assess variability in performance. A similar approach should be take for evaluating  generalizability to unseen cell types).*

Response:

We highly appreciate the careful reading and detailed suggestions by the reviewer.

We agree with the reviewer that leaving out cell types similar to seen cell types cannot  demonstrate the performance of the model. Therefore, we designed the following data splits  and tasks for model evaluation:

1) **We exclude chr8 and chr9 in all cell types from training**. Therefore, gene  expression prediction on chr8 and chr9 is indicative of model's generalization ability,  regardless of the similarity between training and test cell types. We explicitly reported  the model’s performance on chr8 and chr9 in left out cell lines at Line 110-111 and  Extended Data Table 2.

2) **We examined the performance of CREaTor on differentially expressed genes in  K562**. Of the 410 genes differentially expressed in K562 compared to the 19 training

cell types, CREaTor reached a Pearson *r* of 0.68, demonstrating that the model is able  to predict the expression of cell-type specific genes (Extended Data Fig. 2 and Fig. 1d)

3) **We added one analysis demonstrating that the predicted expression profile is  more similar to target cell types inspired by the reviewer's suggestions.** We  compared the similarity of 110 expression profiles from the 19 cell types used for  training, 12 expression profiles of K562 (Supplementary Table 4), as well as the  predicted K562 expression. With dimension reduction and visualization using UMAP,  we found that, predicted K562 expressions are more similar to observed K562  expression profiles, as opposed to other cell types, including those sharing  hematopoietic origins with K562 (Supplementary Figure 2). The result is consistent  for not only genes on chr8 and chr9, but also genes on other chromosomes as well.

4) **We also conducted the leave-one-chromosome-out and leave-one-cell-out  experiment as suggested by the reviewer** (Supplementary Table 2). For the leave one-chromosome-out experiment, the same 19 cell types were used for training, with  K562 held out for test. Each chromosome was excluded from training once, and the  corresponding chromosome in K562 was used for test (Supplementary Table 2a).  Ergo, 22 models were trained for this experiment. The performance of the 22 models  ranges from 0.752 to 0.844, all indicating a significant correlation between prediction  and observed data. This experiment confirms that CREaTor’s performance is not  limited to chr8&9.

For the leave-one-cell-out experiment, chr1-7, chr10-15, and chr17-23 of 19 cell  types are used for training, and chr8-9 in the left-out cell type are used for test. The  correlations between predictions and observations are consistently significant  (Pearson *r* > 0.7), for both chr8 and chr9, in different cell types, in this setting (Supplementary table 2b). This experiment confirms that CREaTor’s performance is  not limited to K562.

Also, we compared the results from leave-one-cell-out to those from leave-K562-out  experiment. In both settings, chr8 and 9 for all cell types were held out. The  performance gap between the two sets of experiments is negligible (Supplementary  Figure 3), indicating CREaTor learns cross-cell type pattern for expression prediction.  Collectively, these results demonstrate the generalization capability of CREaTor.

As our goal is to model cCRE-gene interaction, instead of predicting gene expression, in the  main story, we stick to the setting of leaving K562 out, because K562 has the most abundant  labeled data for the evaluation of enhancer-gene interaction prediction and cis-regulatory  modeling in unseen cell types.

For the same reason, we did not perform extensive comparison between CREaTor and other  methods on gene expression prediction task. However, to claim that CREaTor can model  cCRE-gene interaction accurately, we need to demonstrate that 1) by modeling cCRE-gene  interaction with attention mechanism, CREaTor can make reasonable prediction on gene  expression. 2) by learning universal rules guiding cCRE-gene interactions, CREaTor can  effectively predict cell type-specific genes and genes unseen by the model. Therefore, we  detailed the performance of CREaTor on different gene expression prediction tasks, as in the  first part (*CREaTor predicts cell type-specific gene expression in unseen cell types*) of the  Result section.

*Q3. A second major comment is the lack of model comparison with previous approaches.  For example, MaxATAC (PLoS CompBio 2023), GraphReg (Genome Research 2022), and  ChromDragoNN (Bioinformatics 2019) are three models that use a similar concept: they all  use as input both DNA sequence and epigenomic features and aim to predict gene  expression. It's unclear what the novelty is here. If the novelty is in the model architecture,  then the new architecture should be benchmarked against these previous methods.*

Response:

We sincerely apologize for the lack of clarity and short of comparisons. To improve our  manuscript and address the concerns of the reviewers, we made the following changes:

1) We have modified the Title, Abstract, Background (Line 57-61 and Line 68-76), and  Discussion (Line 329-333) section to stress the novelty and highlights of our study  more clearly. Also, we listed the novelty in the response below.

2) In addition to ABC score and reciprocal distance methods, we included 2 baseline  approaches: Enformer (Avsec et al. 2021) and GraphReg (Karbalaygharch et al. 2022). The comparison is described at Line 183-199, 209-210 and in Figures 2a-b, 2d-e, as  well as Extended Fig. 5. Also, a table highlighting the difference in scopes of the 3  methods (CREaTor, Enformer, and GraphReg) is attached to the response below.

3) In the end, we explained why MaxATAC and ChromDragoNN were not included in the  comparison. In short, neither of these approaches supports zero-shot cCRE-gene  interaction prediction, due to their input size (1000-1024 bp) and training task design.  However, we acknowledge their contributions to sequence-based modeling and gene  regulation study, and explicitly mentioned these studies at Line 407-408.

**Novelty & highlights**

Our study aims to model cis-regulatory patterns and cCRE-gene interactions with improved  accuracy in new cell types without additional training. To achieve this goal, we designed a  training task to predict gene expression based on flanking candidate cis-regulatory elements

(cCREs) up to 2Mb from target genes. With attention mechanisms, our method, CREaTor, is  able to:

• Perform zero-shot cCRE-gene interaction prediction. In other words, no prior  knowledge of cCRE-gene interactions is required for cCRE-gene interaction modeling in training or test data.

• Model cis-regulatory patterns and cCRE-gene interactions in cell types unseen by the  model. In other words, no additional training is required for new cell types. • Model with RNA-seq and ChIP-seq data solely. Most approaches use 3D genomic  data for cCRE-gene interactions prediction, but these data are expensive and are not  readily available, especially for primary cells or tissues.

• Predict cCRE-gene interactions of ultra-long range (more than 1Mb).

We highlighted these points in the abstract, background (Line 57-61 and Line 68-76), and  discussion (Line 329-333) section of the manuscript.

**Enformer & GraphReg**

Both Enformer (Avsec et al. 2021) and GraphReg (Karbalaygharch et al. 2022), trained with  supervised task on gene expression prediction, support zero-shot cCRE-gene interaction  prediction. They both represent the state-of-the-art (SOTA) methods in the domain. However,  there are several drawbacks of these approaches, in comparison to CREaTor, as listed in the  table below:

|  | CREaTor | Enformer | GraphReg |
| --- | --- | --- | --- |
| Zero-shot modeling (Predict cCRE Gene interactions without training  on such data) | ✓ | ✓ | ✓ |
| Long-range (~1Mb) enhancer-gene  modeling | ✓ | × | ✓ |
| Cross-cell type modeling | ✓ | × | ✓, but not rigorously tested  in the original study |
| 3D genomics data-free | ✓ | ✓ | × |

As such, SOTA performance is not expected from these approaches under the rigorous  setting of our study.

Besides, it's important to note that obtaining CAGE data is typically more challenging than  standard gene expression data. Relying on CAGE data for model training could be less cost effective, especially if a model faces difficulties in cross-cell line predictions.

However, we agree with the reviewerthat comparisons are required to demonstrate the value  of CREaTor. Therefore, we included comparisons to Enformer and GraphReg in Figure 2,  Extended Data Fig.5, Line 183-199 and 209-210 in Result section, and Line 57-61 in  Background. The results indicate that CREaTor models enhancer-gene interactions more  accurately in comparison to others.

|  | CREaTor | Enformer | GraphReg |
| --- | --- | --- | --- |
| auROC (overall) | 0.843 | 0.671 | 0.704 |
| auPRC (overall) | 0.667 | 0.366 | 0.294 |

The implementation details of Enformer and GraphReg can be found at Line 573-594 in  Methods section. Also, for reviewer’s convenience, the descriptions are included at the end  of the response.

**ChromDragoNN & MaxATAC**

ChromDragoNN (Bioinformatics 2019) was designed for genome-wide chromatin  accessibility prediction from DNA sequences. It can adapt to gene expression prediction by  replacing the training target from chromatin accessibility to CAGE profiles. However, ChromDragoNN processes sequences of only 1000bp, and its convolutional neural network  architecture restricts it from expanding to long-range interactions. Therefore, it may not be  able to apply to the cCRE-gene modeling task CREaTor focuses on. But it is worth noting that  Enformer’s architecture represents an enhanced version of ChromDragoNN. The  introduction of transformer architecture in Enformer greatly expands its prediction range. Hence, we compared CREaTor to Enformer, instead of ChromDragoNN.

MaxATAC (PLoS CompBio 2023) represents a state-of-the-art tool for TFBS prediction from  ATAC-seq data. Similarly, a major problem with MaxATAC is that it processes sequences of  only 1024bp and is rooted in CNN architecture, which limits it from cis-regulatory events  modeling under the setting of zero-shot learning.

In summary, while both methods can be modified to predict gene expression from DNA  sequences or functional genomics, they lack the ability of extracting global cCRE-genes  interactions under a zero-shot setting due to the architecture constraints. As our goal is to  model cCRE-gene interaction, instead of predicting gene expression, we did not perform  extensive comparison between CREaTor and other methods on gene expression prediction  task. However, we acknowledge their contributions to sequence-based modeling and gene  regulation studies. We mentioned these methods at Line 407-408.

|  | CREaTor | ChromDragoNN | MaxATAC |
| --- | --- | --- | --- |
| Input information | •DNA sequence •Functional  genomics | DNA Sequence | •DNA sequence  •ATAC-seq signal |
| Input sequence range | 2Mb (extendable) | 1000bp | 1024bp |
| Supervised task | Gene expression  prediction | •chromatin  accessibility  •expression of 1630  TFs | 127 models, each  to predict one TF binding site |
| Extract cCRE-gene  interactions under zero shot setting | ✓ | × | × |
| Model multiple cCREs’  activities for one target  gene simultaneously | ✓ | × | × |
| Long-range modeling | ✓ | × | × |
| Cross-cell type modeling | ✓ | × | ✓ |

*Appendix. Details of Enformer and GraphReg*

**Enformer.**

Pre-trained Enformer model was downloaded from https://github.com/deepmind/deepmind research/tree/master/enformer. Genomic sequences flanking genes of interest were prepared  following the original study’s instructions. Gradient x input of candidate enhancers was calculated  following the original study’s instructions. To be pointed out, to simulate prediction tasks in new cell  types, we used the cell-type-agnostic setting during the analysis. More specifically, the gradient was  calculated and aggregated from all human tracks of the model.

**GraphReg**

Epi-GraphReg model was downloaded from https://github.com/karbalayghareh/GraphReg. For  flanking genes of interest, genomic sequences and DNase-seq, H3K27ac, H3K4me3 were prepared  following the original study’s instructions. In the interest of rigorous fairness, we extended the input to  the Epi-GraphReg model to encompass other available histone modifications and transcription factor  (TF) binding sites. Specifically, we incorporated ChIP-seq data for markers including H2AFZ,  H3K4me2, POLR2A, H3K4me1, H3K9ac, POLR2AphosphoS5, H3K9me3, H3K36me3, EP300,  H3K79me2, CTCF, H3K27me3, and H4K20me1 (Supplementary Table 4). For our training set, we  sourced chromosomes from cell lines such as GM12878, B-cell, HeLa-S3, MCF-7, fibroblast-of-dermis,  CD14-positive-monocyte, H1, HepG2, and keratinocyte (Supplementary Table 4), deliberately  excluding chromosomes Chr8, Chr9, and Chr16. The CAGE data for these cell lines were sourced  from ENCODE, as detailed in Supplementary Table 4b. We replaced the 3D genomic data with the  reciprocal of the genomic distance between the cCRE and the TSS. For our validation set, we utilized  chromosomes Chr8, Chr9, and Chr16 from these aforementioned cell lines, and in addition, we  employed the full chromosomal set from the K562 cell line. The computation of saliency and integrated  gradients for candidate enhancers adhered strictly to the protocols provided in the original study.

*Reviewer #2: In this paper, Li et. al. introduce a hierarchical deep neural network, named  CREaTor, developed to predict interactions between candidate cis-regulatory elements  (cCREs) and their target genes. The network incorporates an attention mechanism to model  interactions between genomic elements, even those located 2Mb apart, thereby providing a  more detailed representation of gene regulation involving cis-regulatory programs. In an  evaluation using a distinct cell type, the authors demonstrate that CREaTor surpasses  several of the previously existing methods in encapsulating interactions between cCREs and  genes over differing distance ranges.*

*Overall, I enjoyed reading this paper. The real meat of this study is the innovative, well developed tool that shows promise in overcoming long-standing challenges in regulatory  genomics. The authors make a compelling case that functional data is likely to help CREaTor  generalize to new cell types better than models trained using DNA sequence alone, and  hence I can see this overall strategy becoming widely deployed for the purposes argued by  the authors. The writing is clear though there are multiple typos and grammatical errors that  need to be addressed.*

Response:

We feel great thanks for your professional review and valuable feedback on our article. In  accordance with your insightful suggestions, we have adjusted the manuscript and  conducted additional experiments. Your comments are laid out in *italicized, blue* font, and  specific concerns have been numbered. Our responses are given in regular font, and  changes/additions to the manuscript are highlighted in red.

Furthermore, we have carefully revised any typos and corrected grammatical errors in our  resubmitted manuscript to the best of our knowledge. Since these changes do not affect the  content or structure of the paper, we have not listed them separately but have marked them  in red in the revised manuscript. We apologize for our oversight and appreciate your attention  to detail.

*I have several comments that should be addressed prior to publication:*

*Q1: The authors seek to draw conclusions about the importance of proximal regulatory sites  based on findings that the model maintains substantial predictive power when trained on  windows just 2kb away from the gene. However, much of distal regulation occurs at*

*enhancers within the gene itself, but far away from the gene's promoter. To really support the  author's conclusion as stated, the author should train models using data from just 2kb away  from the transcription start site.*

Response:

We sincerely thank the reviewer for careful reading and apologize for the confusion. We  agree with the reviewer that the transcription start site (TSS), rather than the gene body,  should be used for regulatory elements selection. Upon thorough inspection, we confirm  that the model was indeed trained with data 2kb, 5kb, 10kb, 100kb, or 1Mb away from the  TSS, instead of the gene body. We have corrected the statement at Line 144 and the figure  legend of Extended Figure 3.

*Q2:* The author should probably benchmark against the other attention-based enhancer promoter prediction engine published recently, Enformer (Avsec et. al. 2021).

Response:

We sincerely appreciate the reviewer's constructive comments regarding more  comprehensive benchmarks. As suggested by the reviewer, we conducted additional  experiments to compare CREaTor's performance with two state-of-the-art methods for  enhancer-promoter prediction or enhancer-gene prediction.

1. Enformer (Avsec et al. 2021): Enformer is a cutting-edge model for gene expression  and functional genomic track prediction, which can also be used for enhancer-gene  pair prediction. In the original study, Avsec et al. showcased the outstanding  performance of Enformer on enhancer-gene pair classification using two datasets.  However, since Enformer relies on the reference genome as input, it can only model  cell types present in the training data and is unable to effectively generalize to new  cell types. Additionally, the model is limited to regulatory elements within 200 Kb from  the TSS of target genes.

To compare CREaTor with Enformer, we first extracted genomic regions ± 200kb  flanking the TSS of target genes in the curated enhancer-target gene pair dataset  (Supplementary Table 3). Next, we passed the extracted samples into the pre-trained Enformer model, which was downloaded from  https://github.com/deepmind/deepmind-research/tree/master/enformer. To  simulate prediction tasks in new cell types, we used the cell-type-agnostic setting  during the analysis. More specifically, following the original publication's instructions,

we calculated the gradient from all human tracks of the model. Then, we computed

the gradient x input for the candidate enhancers corresponding to target genes in the  curated dataset. Finally, metrics such as auROC and auPRC on enhancer-gene pair  classification were evaluated for Enformer, using the same settings as CREaTor.

2. GraphReg (Karbalaygharch et al. 2022). The Epi-GraphReg model introduced in this  study predicts gene expression from 1D epigenomic data and 3D genomic structures.  The use of graph attention network and convolutional neural network allows efficient  enhancer-promoter interaction modeling, and the method is also able to generalize  to new cell types, making it a promising tool for cis-regulatory modeling. However, the  model’s dependency on 3D genomic data narrows its applicability, as such data is  not readily available. Consequently, the original study only used four cell types  (GM12878, K562, hESC, and mESC) for modeling.

To compare CREaTor with GraphReg, we retrained Epi-GraphReg with the following  modifications: 1) We trained the model with 9 cell types instead of 1, as in the original  study. For these 9 cell types, they were used for CREaTor training and their CAGE files  can be downloaded from the ENCODE portal (https://www.encodeproject.org/). The  cell types and the accession numbers are listed in Supplementary Table 4c. 2) We  utilized all collected functional data for training cell types, rather than the three tracks  (H3K4me3, H3K27ac, and DNase) mentioned in the original study. 3) We replaced the  3D genomic data with the reciprocal of the genomic distances between the cCRE and  the TSS.

For evaluation, similarly, we extracted 6Mb genomic region surrounding the TSS of  each target gene (Supplementary Table 3) and passed the samples to the model. We  calculated saliency and integrated gradients for candidate enhancers following the  original study’s instructions. As integrated gradients exhibited the best prediction  performance, it was used for comparison across approaches.

As depicted in Fig.2 and Extended Data Fig. 5, the comparison results indicate that CREaTor  more accurately models enhancer-gene interactions in new cell types in the absence of 3D  genomic data (also see below). We have incorporated the corresponding descriptions in the  Background section of the manuscript at Line 57-61, Result section at Line 183-199 and 209- 210, and the Method section at Line 573-579 and Line 581-594).

|  | CREaTor | Enformer | GraphReg |
| --- | --- | --- | --- |
| auROC (overall) | 0.843 | 0.671 | 0.704 |
| auPRC (overall) | 0.667 | 0.366 | 0.294 |

*Q3: The final two browser tracks in Fig 2 have significant rendering issues.* Response:

We apologize for our oversight regarding the rendering issues in Fig 2. To prevent similar  issues, we have double-checked all figures and corrected the rendering problems in the final  two browser tracks of Fig 2. Thank you for bringing this to our attention.

*Q4: The authors write that: "attention weights of CREaTor and quantitative effects of  enhancers on gene expression measured by a CRISPR perturbation study 13 yielded a  spearman ρ of -0.269 (Fig. 2b), implying that CREaTor may capture quantitative effects of  cCREs to genes." It is not intuitive that the correlation should be negative. The author should  clarify here and elsewhere that the measurement they call "effects of enhancers on gene  expression" is actually the effect of enhancer knockdown (and hence, the enhancer would  presumably have the opposite effect).*

Response:

We sincerely appreciate the reviewer’s excellent suggestion and detailed instructions. We  have revised the relevant section according to the reviewer’s recommendation at Line 166- 172, which is also quoted below:

*“In addition, we compared the scores derived from the attention weights of CREaTor  with a quantitative analysis of enhancer effects as described in a previous study^13^. In  this study, the enhancer effect on gene expression was defined as the change in gene  expression upon enhancer knockdown using CRISPR perturbation. Consequently,  the quantitative effect is inversely related to the enhancer activity. In line with this  understanding, we observed a negative correlation, with a Spearman ρ of -0.269,  between the CREaTor scores and the quantitative observations (Fig. 2b), implying that  CREaTor captures quantitative effects of cCREs to genes.”*

Additionally, we have updated the legend of Fig. 2b for clarification. We hope these  modifications address your concerns.

**2^nd^ round**

**Reviewer 1**

The authors have mostly addressed my comments. However, there are still references missing to prior work that is relevant: 1) MaxATAC (PLoS Comp Bio, 2023) is conceptually similar, it takes as input genomic DNA + epigenomic profiles (in this case ATAC-seq) and predicts functional output. 2) DRAGON (Bioinformatics, 2019) takes as input gene expression + DNA sequence and predicts ATAC/TF binding. These two previous model architecture, in addition to GraphReg that is cited now, are designed to perform "zero shot" learning as well. Similarities/differences should still be addressed in Discussion and/or Intro.

**Reviewer 2**

The manuscript has improved in review and I believe it is suitable for publication in genome biology. Congratulations to the authors on a very nice contribution!
